# Supplementary material for: Purification of functional mouse skeletal muscle mitochondria using percoll density gradient centrifugation
Source: BMC Res Notes. 2023 Sep 30;16:243. doi: 10.1186/s13104-023-06519-4 (PMC10544150; doi:10.1186/s13104-023-06519-4)
Supplement: Supplementary file 1 — Supplementary Material 1 [file 13104_2023_6519_MOESM1_ESM.docx]

**Additional File 1: Table S1. Reagents for mitochondrial isolation**

| **Compound** | **Reagent** | **Source, Identifier** |
| --- | --- | --- |
| Chappel-Perry (CP) Buffer I | potassium chloride (100 mM) | Sigma-Aldrich, P9541 |
|  | MOPS (50 mM) | Sigma-Aldrich, M1254 |
|  | EDTA (1 mM) | Sigma-Aldrich, ED2SS |
|  | magnesium sulfate (5 mM) | Sigma-Aldrich, M2643 |
|  |  |  |
| Isolation Medium (IM) | sucrose (225 mM) | Sigma-Aldrich, S0389 |
|  | mannitol (75 mM) | Sigma-Aldrich, M4125 |
|  | EGTA (1 mM) | Sigma-Aldrich, E3889 |
|  | HEPES (5 mM) | Sigma-Aldrich, H3375 |
|  |  |  |
| Isolation Medium (IM) + BSA | fatty acid-free BSA (0.5% and 0.1%) | Sigma-Aldrich, A7030 |
|  |  |  |
| 1X IM w/ 15%, 24% and 40% Percoll | Percoll^®^ density gradient media | Cytiva, 17089102 |
|  |  |  |
